# Redundant and Specific Roles of the ARGONAUTE Proteins AGO1 and ZLL in Development and Small RNA-Directed Gene Silencing

**DOI:** 10.1371/journal.pgen.1000646

**Published:** 2009-09-18

**Authors:** Allison C. Mallory, Annika Hinze, Matthew R. Tucker, Nicolas Bouché, Virginie Gasciolli, Taline Elmayan, Dominique Lauressergues, Vincent Jauvion, Hervé Vaucheret, Thomas Laux

**Affiliations:** 1 Laboratoire de Biologie Cellulaire, Institut Jean-Pierre Bourgin, INRA, Versailles Cedex, France; 2 Faculty of Biology, University of Freiburg, Freiburg, Germany; 3 Station de Génétique et d'Amélioration des Plantes, Institut Jean-Pierre Bourgin, INRA, Versailles Cedex, France; 4 Freiburg Institute of Advanced Studies, University of Freiburg, Freiburg, Germany; National Institute of Genetics, Japan

## Abstract

The *Arabidopsis* ARGONAUTE1 (AGO1) and ZWILLE/PINHEAD/AGO10 (ZLL) proteins act in the miRNA and siRNA pathways and are essential for multiple processes in development. Here, we analyze what determines common and specific function of both proteins. Analysis of *ago1* mutants with partially compromised AGO1 activity revealed that loss of ZLL function re-establishes both siRNA and miRNA pathways for a subset of AGO1 target genes. Loss of *ZLL* function in *ago1* mutants led to increased AGO1 protein levels, whereas *AGO1* mRNA levels were unchanged, implicating ZLL as a negative regulator of AGO1 at the protein level. Since *ZLL*, unlike *AGO1*, is not subjected to small RNA-mediated repression itself, this cross regulation has the potential to adjust RNA silencing activity independent of feedback dynamics. Although *AGO1* is expressed in a broader pattern than *ZLL*, expression of *AGO1* from the *ZLL* promoter restored transgene PTGS and most developmental defects of *ago1*, whereas *ZLL* rescued only a few *AGO1* functions when expressed from the *AGO1* promoter, suggesting that the specific functions of *AGO1* and *ZLL* are mainly determined by their protein sequence. Protein domain swapping experiments revealed that the PAZ domain, which in AGO1 is involved in binding small RNAs, is interchangeable between both proteins, suggesting that this common small RNA-binding domain contributes to redundant functions. By contrast, the conserved MID and PIWI domains, which are involved in 5′-end small RNA selectivity and mRNA cleavage, and the non-conserved N-terminal domain, to which no function has been assigned, provide specificity to AGO1 and ZLL protein function.

## Author Summary

In eukaryotes, short RNAs (21–24 nucleotides long) have broad effects on gene expression through the action of ARGONAUTE (AGO) proteins. The model flowering plant *Arabidopsis thaliana* contains ten AGO proteins, among which AGO1 and ZLL/PNH/AGO10 play a major role in regulating gene expression through small RNA-directed RNA cleavage and translational repression. Here, we address the common and specific effects of *zll* and *ago1* loss of function in Arabidopsis. We show that *zll* mutations lead to increased AGO1 protein levels and suppress a subset of small RNA-directed gene regulatory defects of weak *ago1* mutations. Although AGO1 and ZLL proteins are highly similar in sequence, we show that only the PAZ domain, which in AGO1 is involved in binding small RNAs, can be exchanged between the two proteins. By contrast, the PIWI domain, that is responsible for the RNA cleaving activity of AGO1, the MID domain, which is involved in 5′ nucleotide selection of small RNAs, and the functionally uncharacterized N-terminal domain contribute to their individual functions during small RNA-directed gene regulation and development.

## Introduction

Small RNA-directed gene regulation is a major process in plant development and viral defense [1],[2]. A central component in these pathways is the activity of ARGONAUTE (AGO) proteins, which bind small RNAs and mediate repression of the complementary RNA targets [3],[4]. In *Arabidopsis*, 10 *AGO* genes have been identified [5]. AGO1 [6] associates with numerous microRNAs (miRNAs) and short interfering RNAs (siRNAs) to mediate target repression via mRNA cleavage and inhibition of translation [3],[4],[7]. Binding of AGO1 to miR168, which targets *AGO1* mRNA, establishes a homeostatic *AGO1* regulatory loop [8],[9]. AGO4 and AGO6 function in small RNA mediated chromatin regulation whereas AGO7 associates specifically with miR390 and directs cleavage of the non-protein coding *TAS3* precursor RNA to generate trans-acting short interfering RNAs (tasiRNAs) [5]. Recently, *ZLL* was implicated in miRNA-directed translational inhibition [7] and repression of miR165/166 levels [10].

AGO1 and ZLL protein sequences are highly similar, including the PAZ and MID domains, which bind small RNAs in AGO1 [11], and the PIWI domain, which is required for target mRNA cleavage in AGO1 [3],[4]. By contrast, their N-terminal domains do not display sequence similarities. Both genes differ in their expression patterns and developmental functions. *AGO1* is expressed broadly during plant development, and *ago1* loss-of-function mutants display pleiotropic defects in development and in virus defense [6],[12]. Seedlings of the null allele *ago1-1* form only a few finger-like leaves and about 10% of seedlings lack a shoot meristem. *ago1* mutants are deficient in transgene posttranscriptional gene silencing (PTGS) of *L1 35S:GUS*, a standard reference for systemic sense transgene PTGS in *Arabidopsis* [13], the tasiRNA pathway, and cell autonomous miRNA-directed repression [5]. In contrast to *ago1-1*, the hypomorphic allele *ago1-27*, which expresses an AGO1 protein with reduced mRNA cleavage activity, displays more subtle developmental defects [12].

Expression of *ZLL* is limited to the provasculature and, weaker, to the adaxial (upper) sides of leaves, and ceases as tissue differentiation takes place [14],[15]. In the Landsberg *erecta* (L*er*) accession, *zll* mutant seedlings display differentiated cells or complete organs in place of the shoot meristem stem cells with allele specific penetrance [14]–[16]. Recent studies indicate that *ZLL* function in the provasculature is necessary and sufficient to maintain shoot meristem stem cells during embryogenesis [17]. Furthermore, *ZLL* acts in a sequential manner with *AGO1* during embryogenesis to potentiate *WUSCHEL* (*WUS*) dependent signaling from the stem cell organizer to the stem cells in the developing shoot meristem primordium [17]. *ago1 zll* double mutants of strong alleles result in early embryo arrest, suggesting that both proteins also have redundant activities during early embryo development [14]. Recent findings demonstrated that both proteins function in miRNA-directed repression of *Cu/Zn SUPEROXIDE DISMUTASE 2 (CSD2)* and *SCARECROW-LIKE 6* (*SCL6-IV*) mRNAs and proteins [7]. In contrast to *ago1* mutants, however, *L1* transgene PTGS is not compromised in *zll* mutants [12].

Here, we address specific and overlapping functions of *ZLL* and *AGO1* in development and RNA silencing pathways. Our results indicate that in *ago1* hypomorphic mutants, loss of *ZLL* function restores leaf development and siRNA and miRNA pathways and leads to increased AGO1 protein levels, implicating ZLL as a negative regulator of *AGO1*. Analyses of chimeric gene constructs indicate that the PAZ domain, which is thought to mediate small RNA binding, is exchangeable between both proteins, whereas the MID-PIWI and N-terminal domains appear to contribute to their specific functions.

## Results

### *zll* suppresses leaf defects of *ago1* hypomorphs

**Figure 1:**
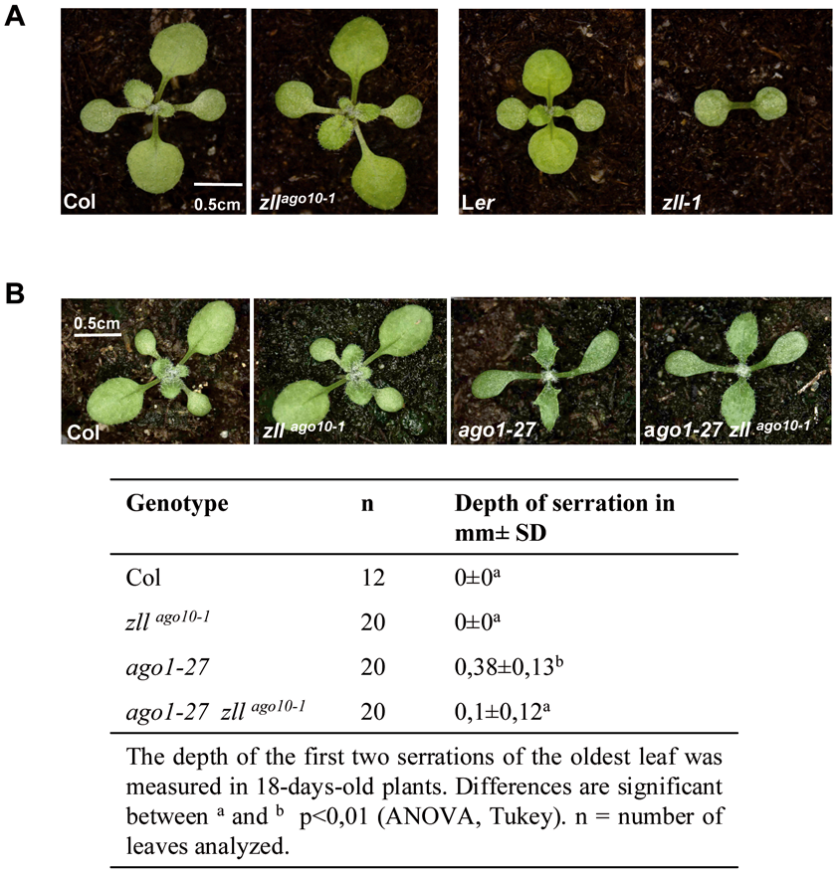
Loss of *ZLL* function partially restores leaf development in *ago1-27*. (A) Null mutations in the *ZLL* gene result in seedlings lacking primary shoot apical meristems in L*er*, whereas no seedling defects are discernable in the Col ecotype. All seedlings are 12 days after germination. Genotypes are indicated. Images are at the same magnification. (B) 18-day-old seedlings of Col, *zll^ago10-1^*, *ago1-27*, and *ago1-27 zll^ago10-1^*. Serration of leaves is reduced markedly in *ago1-27 zll^ago10-1^* compared to *ago1-27*, resulting in a rounded leaf shape similar to Col. Images are at the same magnification. Quantifications of the results with standard deviations are indicated.

To study genetic interactions between *ZLL* and *AGO1*, we analyzed different mutant combinations. Since double mutants of strong *zll* and *ago1* alleles in the L*er* ecotype are embryo lethal [14], we analyzed mutant alleles in the Col ecotype, where *ZLL* loss of function alone does not greatly affect development (Figure 1A and Figure S1), unlike in the L*er* accession, where shoot meristem stem cells are defective [14],[15]. Despite the reduced effect of *zll* mutations in Col compared with L*er*, *ago1-1 zll^ago10-1^* double mutant embryos also arrested at the late globular stage with defects in cell division, cell elongation, and expression of both *WOX5* and *WUS* genes, which mark root and shoot stem cell niches, respectively (Figure S2). None of these effects were observed in any single mutant, indicating redundant functions of *ZLL* and *AGO1*. To avoid embryo lethality obtained in double mutants with the null allele *ago1-1* [6] and to enable the analysis of genetic interactions during postembryonic development, we used the hypomorphic *ago1-27* mutant in combinations with *zll^ago10-1^* and *zll^ago10-3^* alleles. *ago1-27* mutants are defective in small RNA-directed regulation [9],[12] and, in contrast to the severe growth and developmental defects of *ago1-1*, display increased leaf margin serration, reduced leaf width, abnormal flower phyllotaxis, and reduced fertility compared to wildtype [12]. By contrast, seedlings of *zll^ago10-1^* and *zll^ago10-3^* single mutants did not display any noticeable leaf defects (Figure 1 and Figure S1A) [18] and only infrequently a defective shoot meristem (0.2%, n>1000) [14],[15]. Surprisingly, *ago1-27 zll^ago10-1^* and *ago1-27 zll^ago10-3^* double mutants revealed that both *zll* mutations partially suppressed the increased leaf margin serration of *ago1-27* (Figure 1B and Figure S1A), rather than enhancing it as we expected for two related AGO proteins involved in RNA silencing. By contrast, neither the phyllotaxis nor the fertility defects of *ago1-27* were restored by the *zll* mutations (data not shown).

### *zll* mutations restore transgene PTGS and miRNA-mediated gene silencing in hypomorphic *ago1* mutants

**Figure 2:**
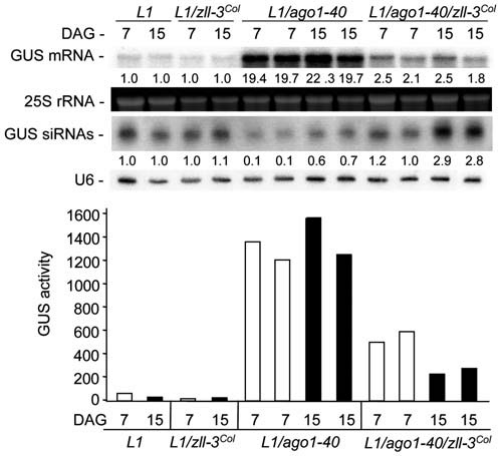
*zll* mutations promote *L1* PTGS when AGO1 function is partially compromised. RNA gel blot analyses of *GUS* mRNA and *GUS* siRNA accumulation and GUS protein activity in 7- and 15-day-old *L1* control, *L1/zll-3* ^Col^, *L1/ago1-40*, and *L1/ago1-40 zll-3* ^Col^ seedlings. Two biological replicates of *L1/ago1-40* and *L1/ago1-40 zll-3* ^Col^ are shown for each time point. *25S* rRNA and *U6* RNA were used as loading controls for mRNAs and siRNAs, respectively. Normalized values of *GUS* mRNA to *25S* rRNA and *GUS* siRNAs to *U6* RNA (with control *L1* 7- and 15-day-old seedling levels set at 1.0) are indicated. DAG (days after germination).

To study *ZLL* and *AGO1* interactions at the level of RNA silencing, we first analyzed PTGS of the *L1 35S:GUS* transgene. Our previous studies indicated that PTGS of the *L1 35S:GUS* transgene was compromised in *sgs3*, *rdr6*, *hen1*, and *ago1* mutants but not in *zll* single mutants [12],[19],[20]. The newly identified *ago1-40* EMS mutation causes an A to V amino acid change at position 863 of the protein, resulting in increased mRNA levels and protein activity and decreased siRNA levels for the *L1 35S:GUS* transgene (Figure 2 and Table S1). Unlike previously identified *ago1* mutations that impair *L1* PTGS with 100% efficiency, about 50% of *ago1-40* adult plants at each generation had triggered PTGS, allowing us to test whether *zll* mutations affected *L1* PTGS in *ago1-40*. To avoid any potential interference between the 35S promoters embedded in the T-DNA of the available insertional *zll* mutants in Col and the *L1 35S:GUS* transgene [21], we backcrossed five times to *L1* the EMS-induced *zll-3* mutant, which was isolated in the L*er* accession [15]. *L1/zll-3* ^Col^ had similar *GUS* mRNA levels, protein activity and siRNA levels as silenced *L1* controls (Figure 2) [12].

*GUS* mRNA levels in *L1/ago1-40 zll-3^Col^* double mutants were reduced in comparison to *L1/ago1-40* mutants to nearly the level of silenced *L1* controls (Figure 2). This increase in *L1* silencing in the double mutant correlated with increased levels of *GUS* siRNAs. Seven days after germination (DAG), *GUS* siRNA levels were more than 10-fold higher than in *L1/ago1-40* mutants, reaching levels comparable to silenced *L1* controls 7 DAG, and by 15 DAG even exceeding *L1* control levels (Figure 2). Thus, loss of *ZLL* function restored *L1* gene silencing compromised in *ago1-40*.

**Figure 3:**
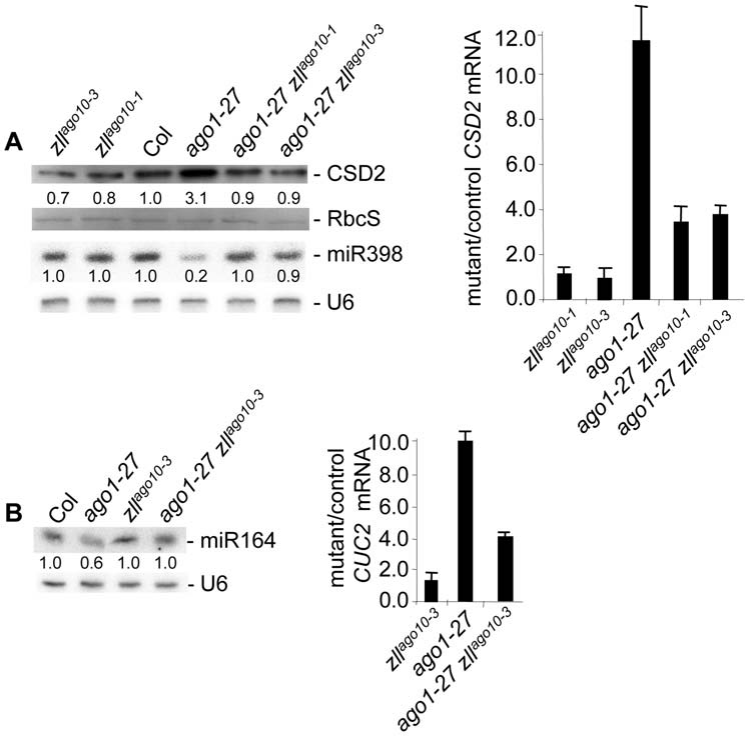
*zll* mutations restore miR398- and miR164-directed silencing of *CSD2* and *CUC2*, respectively, when *AGO1* function is partially compromised. (A) Immuno blot of CSD2 protein in seedlings of the indicated mutant lines and their Col control in the absence of CuSO_4_. Coomassie blue–stained RUBISCO small subunit (RbcS) serves as a loading control. Normalized values of CSD2 protein to RbcS controls (with Col controls set at 1.0) are indicated. RNA gel blot analysis of miR398 in seedlings of the indicated mutant and control lines in the absence of CuSO_4_. Normalized values of miR398 to *U6* RNA (with Col controls set at 1.0) are indicated. miR398 is induced by copper starvation and is undetectable in the presence of copper [34] (Figure S3). Quantitative RT-PCR of *CSD2* mRNA in seedlings of the indicated mutant lines in the absence of CuSO_4_. Average values of three technical replicates were normalized to *EF1a* control values and standard deviations are shown. (B) RNA gel blot analysis of miR164 in the indicated mutant and control lines. Normalized values of miR164 to *U6* RNA (with Col controls set at 1.0) are indicated. Quantitative RT-PCR of *CUC2* mRNA in the indicated mutant lines. Average values of three technical replicates were normalized to *EF1a* control values, and standard deviations are shown.

To address whether *zll* mutations also were able to restore the miRNA pathway in *ago1* hypomorphs, we analyzed miRNA levels and miRNA-regulated target genes in the *ago1-27 zll^ago10^* double mutants. miR398 levels were reduced and *CSD2* mRNA and protein levels were substantially elevated in *ago1-27* compared to *zll^ago10-3^* and *zll^ago10-1^* single mutants and wildtype (Figure 3A and Figure S3). By contrast, in both *ago1-27 zll^ago10-3^* and *ago1-27 zll^ago10-1^* double mutants, *CSD2* mRNA and protein levels were reduced and miR398 levels were elevated, compared to *ago1-27* alone (Figure 3A and Figure S3). The *zll^ago10-3^* mutation also restored miR164 accumulation and miR164-directed *CUC2* silencing to wildtype levels in the *ago1-27* background (Figure 3B). To extend our investigation to the whole-genome level, a transcriptome analysis was performed using Col wildtype, *ago1-27*, *zll^ago10-1^* and *ago1-27 zll^ago10-1^*. Among 46 miRNA targets that were elevated in *ago1-27* compared to wildtype but which were not affected in *zll^ago10-1^* single mutants, 19 were reduced completely or partially to wildtype levels in the *ago1-27 zll^ago10-1^* double mutant (Table S2). Taken together, loss of ZLL function restored *L1* PTGS and silencing of approximately half of the miRNA targets deregulated in *ago1-27*.

### *zll* mutations enhance AGO1 protein accumulation in hypomorphic *ago1* mutants

**Figure 4:**
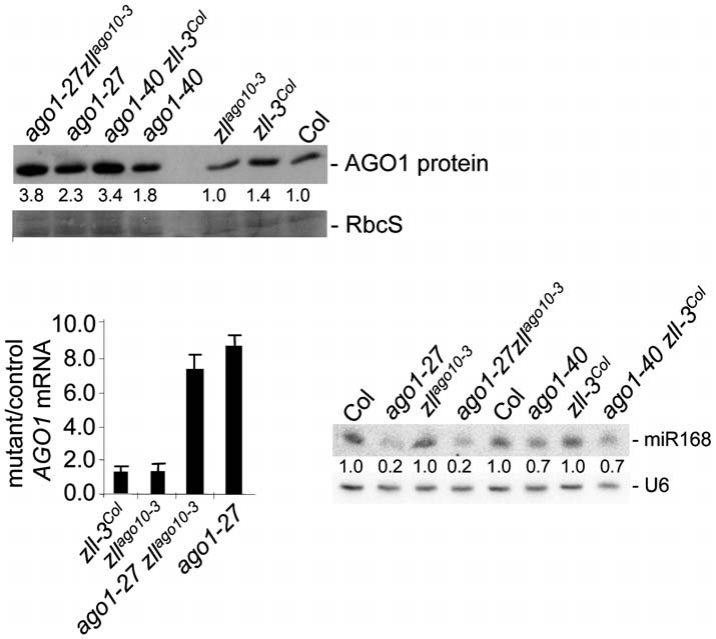
Loss of ZLL function leads to increased AGO1 protein levels in *ago1* hypomorphic mutants. Immuno blot of AGO1 protein in seedlings of the indicated mutant lines and their Col control. Coomassie blue–stained RUBISCO small subunit (RbcS) serves as a loading control. Normalized values of AGO1 protein to RbcS controls (with Col controls set at 1.0) are indicated. Quantitative RT-PCR of *AGO1* mRNA in seedlings of the indicated mutant lines. Average values of three technical replicates were normalized to *EF1a* control values, and standard deviations are shown. RNA gel blot analysis of miR168 in seedlings of the indicated mutant and control lines. Normalized values of miR168 to *U6* RNA (with Col controls set at 1.0) are indicated.

The suppression of developmental, *L1* silencing and miRNA pathway defects in hypomorphic *ago1* mutants by *zll* mutations raised the question whether *ZLL* might be a negative regulator of *AGO1*. To test this hypothesis, we compared *AGO1* mRNA and protein levels in *ago1*, *zll* and *ago1zll* double mutants. AGO1 protein levels were increased in both *ago1-27 zll^ago10-3^* and *ago1-40 zll-3^Col^* double mutants compared to the corresponding *ago1* single mutants (Figure 4 and Figure S4). *AGO1* mRNA and miR168 levels, however, were not significantly different (Figure 4). This indicates that *ZLL* is a negative regulator of *AGO1* at the protein level, consistent with the role of *ZLL* in translational inhibition [7].

### Protein sequence and specific expression patterns determine the functional differences between *AGO1* and *ZLL*

**Figure 5:**
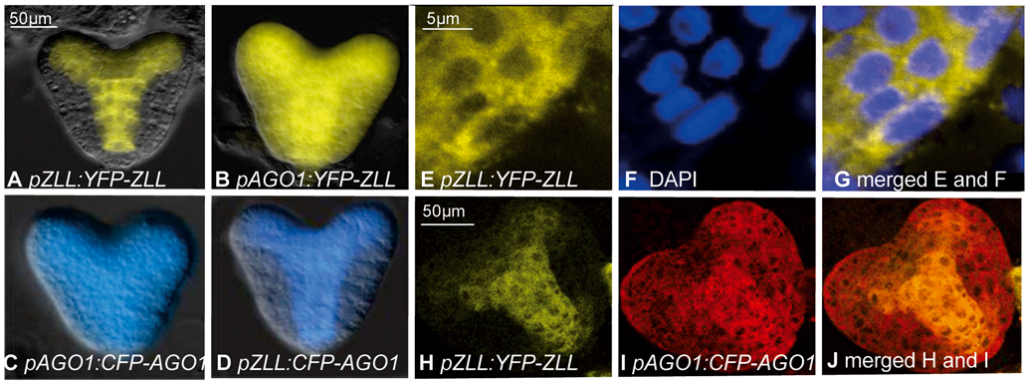
Expression of AGO1 and ZLL reporter proteins in heart stage *Arabidopsis* embryos. (A–D) Epifluorescence images of Col seedlings showing expression of *YFP-ZLL* and *CFP-AGO1* from the respective endogenous promoter and with swapped promoters as indicated. Images (A–D) are at the same magnification. (E–G) Confocal images of heart stage embryo cells expressing *pZLL:YFP-ZLL* outside the nuclei, which are marked by blue DAPI staining. (H–J) Confocal images showing overlapping expression of *pZLL:YFP-ZLL* (yellow) and *pAGO1:CFP-AGO1* (colored red for clarity).

To determine whether the specific effects of *ago1* and *zll* mutations could be explained by the expression patterns of *AGO1* and *ZLL*, we first compared the expression patterns of *pZLL:YFP-ZLL* and *pAGO1:CFP-AGO1* reporter genes. Both reporter constructs rescued the corresponding mutants, indicating that the fusion proteins are functional (Table S3) [17]. *YFP-ZLL* and *CFP-AGO1* proteins were detected in a largely overlapping punctuate pattern outside the nucleus of expressing cells (Figure 5E–5J). As previously reported, *pZLL:YFP-ZLL* is initially expressed throughout the embryo, but becomes limited to provascular strands and the adaxial side of the cotyledons at about the globular stage (Figure 5A) [17]. By contrast, *pAGO1:CFP-AGO1* is expressed in the whole embryo with the strongest signal in the provascular cells from globular stage to early torpedo stage (Figure 5C). Thus, *ZLL* and *AGO1* expression patterns overlap partially, with the *AGO1* expression pattern being broader than the one of *ZLL*, in agreement with mRNA localization results [14].

**Figure 6:**
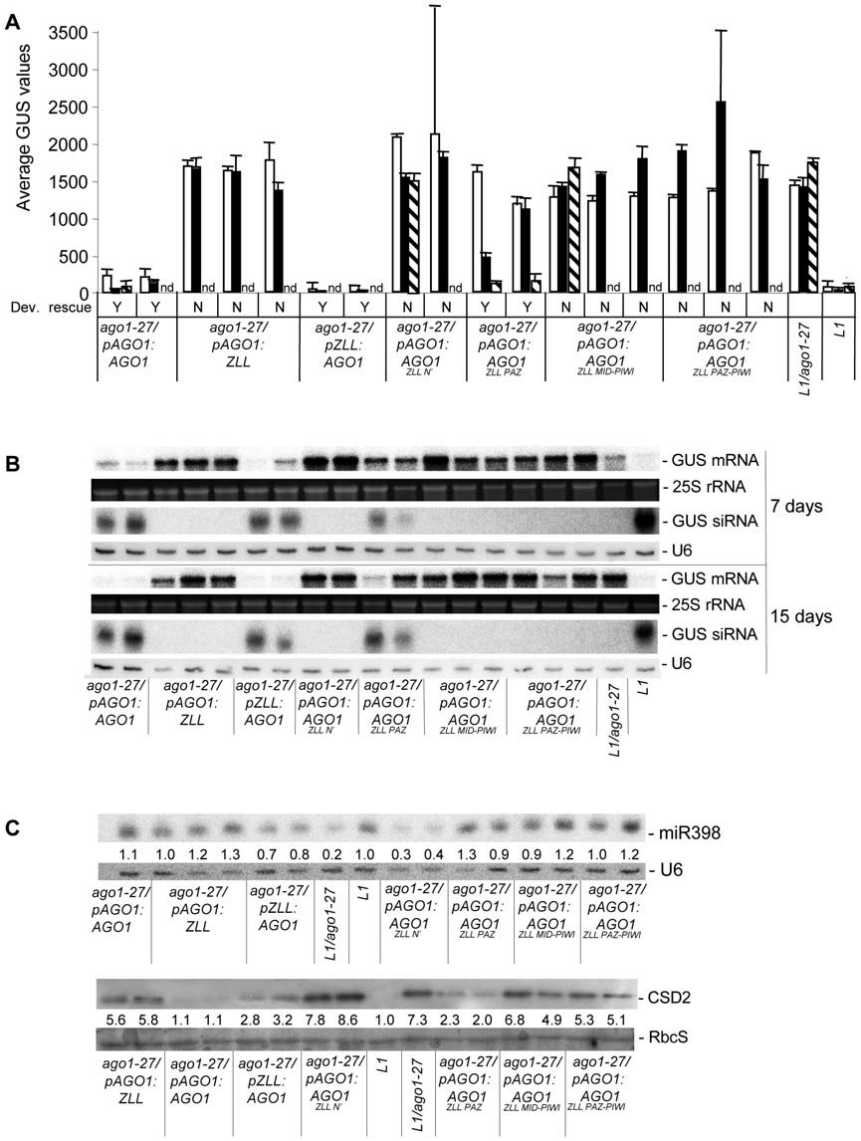
Restoration of *L1* PTGS in *ago1-27* by AGO1^ZLL^ chimeras. (A) Average GUS protein activity (nmol MU/min/µg protein) in 7-day-old (white bars), 15-day-old (black bars), and 21-day-old (hashed bars) seedlings of *L1* and *L1/ago1-27* and *L1/ago1-27* harboring the different chimeric *AGO1^ZLL^* genes as indicated. For all constructs, 2–3 independent homozygote T3 lines and a minimum of 12 plants per line were analyzed. Standard deviations are shown. nd, not determined in 21-day-old seedlings. Dev. rescue, Developmental rescue; Y, yes; N, no. (B) Northern blot analyses of *GUS* mRNA and siRNA accumulation in 7- and 15-day-old seedlings of *L1*, *L1/ago1-27*, and *L1/ago1-27* lines harboring the different chimeric *AGO1^ZLL^* genes as indicated. At least two biological replicates are shown for each chimera at each time point. *25S* rRNA and *U6* RNA were used as a loading control for mRNAs and siRNAs, respectively. (C) Northern blot analyses of miR398 and immuno blot analysis of CSD2 protein accumulation in 15-day-old seedlings of *L1*, *L1/ago1-27* and *L1/ago1-27* lines harboring the different chimeric *AGO1^ZLL^* genes, as indicated. Normalized values of miR398 and CSD2 protein to *U6* and RbcS controls, respectively, (with *L1* controls set at 1.0), are indicated. At least two biological replicates are shown for each chimera. *U6* RNA and coomassie blue–stained RUBISCO small subunit (RbcS) serve as loading controls.

To evaluate the significance of the broad *AGO1* expression pattern, we expressed *AGO1* from the *ZLL* promoter and found that *pZLL:AGO1* by and large restored development of *ago1-1* (Table 1 and Figure S5) and *ago1-27* (data not shown) mutants and also *L1* PTGS in *ago1-27* (Figure 6A–6B). However, miR398 accumulation and *CSD2* silencing were only partially restored in *ago1-27/pZLL:AGO1* (Figure 6C). These results suggest that limiting expression of *AGO1* to the *ZLL* region is sufficient to provide most *AGO1* functions in development and RNA silencing. Nevertheless, expression in cells outside the *ZLL* pattern is required to completely restore AGO1 activity.

**Table 1:** Restoration of leaf formation in *ago1-1* seedlings expressing *AGO1/ZLL* chimeras.

Plant Genotype	n	Leaf Numbers ±SD
Col	8	7.6±0.9^a^
*ago1-1*	54	0.04±0.2^b^
*ago1-1/pAGO1:AGO1*	15	7.1±0.9^a^
*ago1-1/pAGO1:AGO1 ^ZLL PAZ^*	27	7.1±1.0^a^
*ago1-1/pZLL:AGO1*	22	7.3±0.7^a^
*ago1-1/pAGO1:AGO1 ^ZLL PAZ-PIWI^*	15	3.0±1.0^c^
*ago1-1/pAGO1:AGO1 ^ZLL MID-PIWI^*	22	3.2±1.3^c^
*ago1-1/pAGO1:AGO1 ^ZLL N′^*	27	3.3±0.9^c^
*ago1-1/pAGO1:ZLL*	59	0.1±0.4^d^

Leaf numbers were counted 20 days after germination. Plants were germinated on 0.5×MS medium and then transferred to soil.

a,b,c,d Differences are significant between ^a,b,c^ and ^d^ , P<0.01, respectively (ANOVA,Tukey).

n, number of analyzed plants from 2–3 independent primary transformants. SD, standard deviation.

Next, we addressed whether differences within ZLL and AGO protein sequences are responsible for differences in their functions by analyzing whether AGO1 could replace ZLL and vice versa. *AGO1* expression from the *ZLL* promoter (*pZLL:AGO1*) rescued shoot meristem formation in the *zll-1* mutant in the majority of cases (Table 2). By contrast, expression of *ZLL* from the *AGO1* promoter (*pAGO1:ZLL*) in the strong *ago1-1* allele resulted only in a slight reduction of leaf radialization compared to untransformed *ago1-1* (Figure S6), but did not rescue any other developmental defect. Furthermore, in the *ago1-27* hypomorph, *pAGO1:ZLL* was unable to rescue altered flowering time, reduced rosette size (Figure S7), *L1* PTGS (Figure 6A and 6B) or *CSD2* regulation (Figure 6C). Thus, whereas AGO1 can largely replace ZLL function in stem cell development, ZLL appears unable to efficiently replace the developmental, miRNA and PTGS functions of AGO1. Intriguingly, although *pAGO1:ZLL* did not restore *CSD2* silencing in *ago1-27*, it fully restored miR398 accumulation to wildtype levels (Figure 6C). These results suggest that the intrinsic differences of AGO1 and ZLL proteins determine their specific contribution to small RNA and development pathways.

**Table 2:** Frequency of shoot meristem defects in *zll-1* seedlings expressing *ZLL/AGO1* chimeras.

Plant Genotype	n	Seedlings Lacking a Shoot Meristem (%) ±SD
*zll-1*/empty vector	7	81±8.9^a^
*zll-1/pZLL:ZLL*	7	0.0±0.0^b^
*zll-1/pZLL:AGO1*	14	22±18* c
*zll-1/pZLL:ZLL ^AGO1 PAZ^*	8	2.1±4.2^b^
*zll-1/pZLL:ZLL ^AGO1 MID-PIWI^*	8	2.5±7.1^b^
*zll-1/pZLL:ZLL ^AGO1 N′^*	8	64±6.5^a^

The fraction of 12-day-old seedlings lacking a shoot meristem is indicated based on >50 seedlings for each transgenic line.

*: Seedlings displaying a phenotype suggestive of *AGO1* cosuppression are not included.

a,b,c,d Differences are significant between ^a, b^, and ^c^, respectively (ANOVA,Tukey).

n, number of independent transgenic lines analyzed. SD, standard deviation.

### The PAZ domain, but not the ZLL MID-PIWI- or N-terminal domains, is exchangeable between ZLL and AGO1 proteins

To address whether and if any ZLL and AGO1 protein domains have similar functions, we analyzed the ability of chimeric proteins composed of AGO1 and ZLL domains to rescue the respective mutant defects. As expected from the *pZLL:AGO1* result, most chimeric ZLL^AGO1^ proteins (where one AGO1 protein domain was embedded in a ZLL protein backbone) driven from the *ZLL* promoter rescued shoot meristem formation of the *zll-1* mutant (Table 2). The marked exception was the AGO1 N-terminal domain (*pZLL:ZLL^AGO1 N′^*) that could not efficiently replace the corresponding ZLL N-terminal domain (Table 2). This finding was unexpected since the complete AGO1 protein largely replaced ZLL, and might indicate that the function of the N-terminal domain is sensitive to the correct protein context.

**Figure 7:**
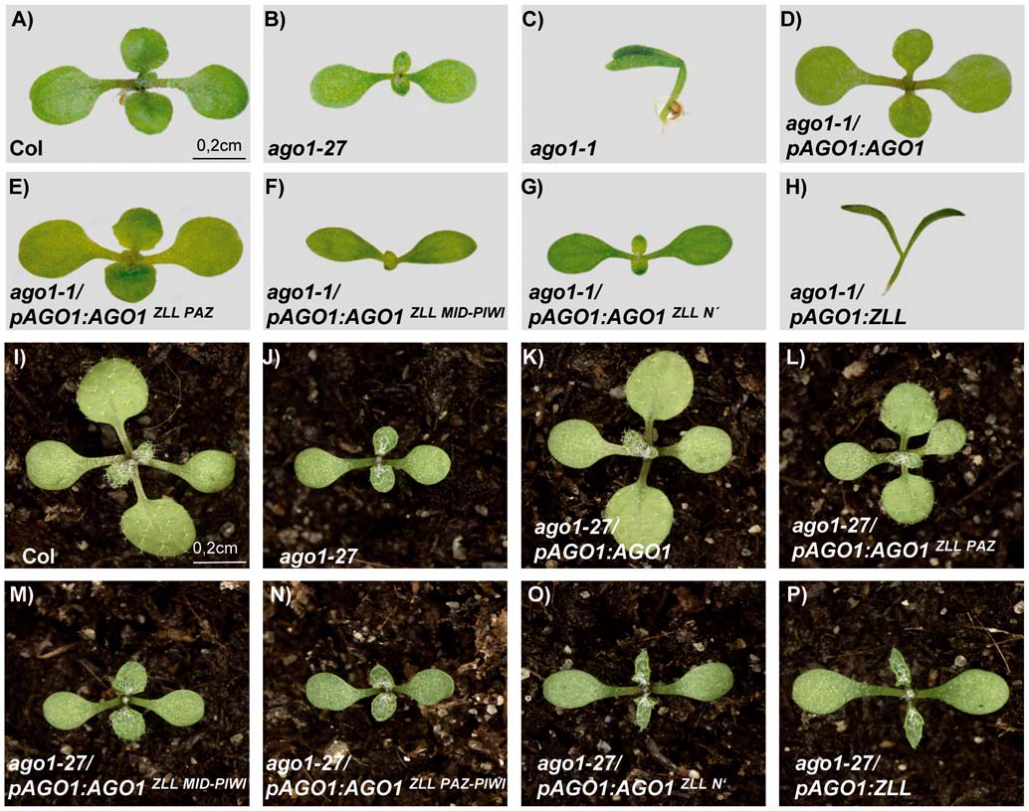
Rescue of *ago1* seedling defects by AGO1^ZLL^ chimeras. (A–C,I,J) Wildtype Col and *ago1* mutant controls. (D–H) *ago1-1* plants harboring chimeric constructs as indicated. (K–P) *ago1-27* plants harboring chimeric constructs as indicated. Images are at the same magnification. 12-days-old plants are shown.

On the converse, only the ZLL PAZ domain within the AGO1 backbone (*pAGO1:AGO1^ZLL PAZ^*) efficiently rescued developmental defects not only of the *ago1-27* hypomorph (Figure 7L and Figure S7) but also of the null *ago1-1* allele (Figure 7E, Table 1, and Figure S5). The ZLL PAZ domain also largely restored *L1* PTGS and *GUS* siRNA accumulation, and *CSD2* silencing and miR398 accumulation in *ago1-27* (Figure 6). PTGS restoration, however, was delayed compared to the developmental rescue (Figures 6A and 7), consistent with previous findings that PTGS is more sensitive than development to compromised AGO1 activity [12]. By contrast, replacing the N-terminal or MID-PIWI domains of AGO1 with the corresponding ZLL regions (*pAGO1:AGO1^ZLL N′^* and *pAGO1:AGO1^ZLL MID-PIWI^*) only restored bilateral leaf development but not sterility of *ago1-1* mutants (Figure 7C, 7F, and 7G, Table 1, and Figure S5), or any developmental defects of *ago1-27* mutants (Figure 7M and 7O and Figure S7). In addition, neither the N-terminal domain nor the MID-PIWI domains of ZLL were able to restore *L1* PTGS and *GUS* siRNA accumulation or *CSD2* silencing in *ago1-27* (Figure 6). Since previous studies have indicated that PAZ, MID and PIWI domains function together in small RNA binding [11],[22],[23], we constructed a *pAGO1:AGO1^ZLL PAZ-PIWI^* chimera where the *AGO1* genomic region containing PAZ, MID and PIWI domains was replaced by the corresponding *ZLL* genomic sequence (Figure S8). *pAGO1:AGO1^ZLL PAZ-PIWI^* resulted in similar effects as *pAGO1:AGO1^ZLL MID-PIWI^* (Table 1, Figures 6 and 7, and Figure S7). This suggested that the failure of the ZLL MID-PIWI domains to restore the majority of *ago1* defects was not due to an incompatibility with the AGO1 PAZ domain or the disruption of the region connecting the PAZ and PIWI domains. Notably, although the *pAGO1:AGO1^ZLL PAZ-PIWI^* did not rescue *CSD2* silencing, it restored miR398 accumulation in *ago1-27* (Figure 6C).

In summary, these results indicate that the ZLL PAZ domain has the capacity to fulfill AGO1 functions in development, the miRNA pathway, and PTGS whereas the ZLL N-terminal and MID-PIWI domains are largely incompatible with AGO1 activity.

## Discussion

As part of the small RNA-directed RNA silencing machinery, the closely related ZLL and AGO1 proteins fulfill important roles during *Arabidopsis* development. Previous studies of mutant phenotypes indicate the presence of both, redundant, specific, and even opposite functions of ZLL and AGO1. Here, we investigate the diversity of ZLL and AGO1 functions and show that ZLL acts as a negative regulator of AGO1, and that the activities of the two proteins are determined by both functionally equivalent and distinct domains.

### Redundant functions of ZLL and AGO1

We find that double mutant combinations of strong *zll* and *ago1* alleles are embryo lethal with strong patterning defects, revealed by abnormal expression of marker genes for the shoot and root meristem stem cell niche. This indicates that ZLL and AGO1 have a significant set of redundant functions required during early embryo development, in line with previous reports [14]. Although we have been unable to directly determine the small RNAs bound to ZLL due to the instability of the ZLL protein, we present several lines of indirect evidence suggesting that ZLL and AGO1 have partially redundant functions in small RNA-mediated silencing, and that ZLL domains are capable of binding a subset of small RNAs bound by AGO1: (1) Our protein domain swapping experiments indicate that the PAZ domain, which has been shown to bind small RNAs in several AGO proteins [11], is interchangeable between ZLL and AGO1, providing fully active proteins, (2) miR398 accumulation is restored to wildtype levels in an *ago1* hypomorph by expression of *pAGO1:ZLL*, *pAGO1:AGO1^ZLL PAZ^*, *pAGO1:AGO1^ZLL MID-PIWI^* and *pAGO1:AGO1^ZLL PAZ-PIWI^* chimeras, and (3) both AGO1 and ZLL negatively regulate AGO1.

### Opposing effects of *ago1* and *zll* mutations

In addition to redundant functions of AGO1 and ZLL, our results using hypomorphic *ago1* alleles to circumvent embryo lethality demonstrate opposing effects of *ago1* and *zll* mutations. First, loss of *ZLL* function re-establishes both PTGS of the *L1* transgene and miRNA-directed repression of a subset of target mRNAs deregulated in *ago1-27*, including miR398- and miR164 directed repression of their *CSD2* and *CUC2* targets, respectively. Furthermore, we observe partial suppression of hypomorphic *ago1* leaf serration defects by *zll* mutations, which could be due to the partial re-establishment of miR164-directed *CUC2* regulation in *ago1 zll* double mutants (Figures 1 and 3 and Figure S1) [24]. These opposite effects of *ago1* and *zll* mutations are consistent with recent findings showing that mRNAs of leaf polarity-related HD-ZIP transcription factors and the corresponding miR165/166 are affected oppositely in *zll* and in *ago1* single mutants (S. Bosca and T.L. unpublished) [9],[10],[25]. A plausible explanation for the restoration of developmental and RNA silencing defects caused by reduced AGO1 activity is provided by our finding that loss of ZLL activity results in upregulation of AGO1 protein levels in *ago1-27*. This negative regulation of AGO1 by ZLL suggests that homeostasis of AGO activity involves cross-regulation between different AGO proteins, which in the case of ZLL affects AGO1 protein but not mRNA levels, consistent with the recent implication of *ZLL* in translational repression [7]. Importantly, since *ZLL* expression itself is not a target of small RNA-mediated repression whereas *AGO1* is [9],[26], ZLL has the potential to provide an input into RNA silencing activity that is independent of negative feedback dynamics and thus might serve to mediate, for example, developmental tuning of RNA silencing.

However, silencing of all miRNA targets deregulated in *ago1-27* is not restored by the absence of *ZLL* function. One possible explanation is that upregulation of AGO1 protein levels in *ago1 zll* double mutants does not restore AGO1 activity completely to wildtype levels, which might be required for efficient silencing of a subset of target genes. Alternatively, since the miRNA pathway is cell autonomous [27],[28], the re-establishment of silencing of miRNA targets is expected to be limited to tissues where *AGO1* and *ZLL* are co-expressed but will not take place in tissues where only *AGO1* is expressed. This explanation is consistent with the *pZLL:AGO1* analysis, where limiting *AGO1* expression to the *ZLL* domain in *ago1* mutants restored systemic *L1* PTGS but did not fully restore miR398 accumulation and *CSD2* regulation. Future experiments comparing *AGO1*, *ZLL* and miRNA tissue-specific expression will help to discriminate between these two possibilities.

### Determinants of specific AGO1 and ZLL activities

Even though the sequences of ZLL and AGO1 proteins are closely related, the corresponding single mutants display different developmental defects. The pleiotropic *ago1* mutants are defective in leaf morphology, general growth, and fertility, whereas *zll* mutants in the L*er* accession display specific developmental defects in shoot apical meristem, flower, and silique development with allele specific penetrance. In contrast to the interchangeable PAZ domain, the non-conserved N-terminal domains, for which a function has yet to be assigned, cannot be exchanged between AGO1 and ZLL without loss of activity. Similarly, exchange of the MID and PIWI domains, which in AGO1 have been shown to provide selectivity for small RNAs possessing a 5′ U [22] and to function as a slicer domain that cleaves mRNA, respectively [3],[4], also cannot provide fully active proteins. This indicates that these domains contribute to functional differences. It is possible that the inability of the ZLL MID-PIWI fragment to replace the AGO1 domains reflects different preferences for 5′ nucleotide selectivity. Since the consensus amino acid residues essential for mRNA cleavage in several AGO1 proteins [29] are present in the ZLL PIWI domain, it is conceivable that both AGO1 and ZLL have the capacity to silence via mRNA cleavage and translational inhibition, but that each protein has a different preference for one of the two mechanisms, in line with recent findings [7].

Future dissection of AGO1 and ZLL properties will help to reveal how the interplay between AGO1 and ZLL proteins influences silencing specificity and efficiency in development.

## Materials and Methods

### Plant material

The following mutants in the Col ecotype have been described previously: *ago1-1* [6], *ago1-27* [12], and *zll^ago10-1^* [18]. *ago1-27 zll^ago10-3^* mutants were generated using the *zll^ago10-3^* mutant (SALK_519738), which expresses a mis-spliced transcript that lacks part of exons 13 and 14 creating a frame-shift mutant with reduced levels of *ZLL* mRNA (Figure S1B). *ago1-40* displays developmental defects (data not shown) similar in range, although much milder than *ago1-27* [12]. *zll-1*, *zll-3*, and *zll-15* mutants were isolated in the L*er* accession as described [15]. Plants on soil were grown as described previously [30]. Plants on agar plates where grown on 1/2×MS supplemented with Gamborg vitamins (Sigma) and 10 g/l saccharose if indicated.

### RNA analysis and GUS activity quantification

For RNA gel blot analyses, frozen tissue was homogenized in a buffer containing 0.1 M NaCl, 2% SDS, 50 mM Tris-Hcl (pH 9), 10 mM EDTA (pH 8) and 20 mM beta mercaptoethanol and RNAs were extracted two times with phenol. RNA gel blot analyses and quantification of GUS activity were performed as described [31]. Hybridization signals were quantified using a Fuji phosphor imager and normalized to a *U6* oligonucleotide probe for miRNA gel blot analyses. *GUS* mRNA and GUS activity analyses were performed on the aerial parts of 7-day-, 15-day- and 21-day-old seedlings grown on Bouturage media (Duchefa) in 16 hours light, 8 hours dark at 22°C. For the *CSD2* and miR398 analyses, seeds were germinated on media [32] without sucrose in both the presence and absence of 0.5 µM CuSO_4_, and plants were grown in 16 hours light, 8 hours dark at 22°C for 12 days at which time the aerial portion of the seedlings were harvested and homogenized in liquid nitrogen. For the *CUC2*, miR164, *AGO1* and miR168 analyses, plants were grown for 10 days on media [32] in the presence of 0.5 µM CuSO_4_. For cDNA synthesis, RNAs were extracted with the Plant RNeasy kit (Qiagen), treated with DNAseI (Invitrogen) and l µg of DNA-free RNA was reverse transcribed with oligo-dT (Invitrogen). Quantitative real time (QRT)-PCR, was performed on a MasterCycler ep realplex (Eppendorf) with the RealMAster SYBR ROX mix (5PRIME) according to the manufacturer's protocol. Each reaction was performed on 5 µl of 1∶60 dilution of the cDNA and synthesized in a 20 µl total reaction. Specific oligonucleotide pairs were:

*EF1a*: 5′- CTGGAGGTTTTGAG GCTGGTAT -3′,

5′- CCAAGGGTGAAAGCAAGAAGA -3′;

*CSD2*: 5′- CAGAAGATGAGTGCC GTCATGCGG -3′,

5′- CCGAGGTCATCCTTAAGCTCGTG -3′;

*CUC2*: 5′- GCA CCAACACAACCGTCACAG -3′,

5′- GAATGAGTTAACGTCTAAGCCCAAGG-3′ and

*AGO1*: 5′- AAGGAGGTCGAGGAGGGTATG -3′,

5′- CAAATTGCTGAGCCAGAACAG -3′. The reactions were incubated at 95°C for 2 minutes to activate the hot-start recombinant Taq DNA polymerase, followed by 45 cycles of 15 seconds (s) at 95°C, 15 s at 60°C and 20 s at 68°C to ensure primer extension and to measure the fluorescence signal. The specificity of the PCR amplification procedures was checked with a heat dissociation protocol (from 60°C to 95°C) after the final cycle of PCR. The efficiencies of the primer sets were evaluated by performing QRT-PCR on several dilutions of a mix of the different strands. The results obtained on the different genotypes were standardized to the expression level of *EF1a*. For microarray analyses, RNAs were extracted using the RNeasy Plant Mini Kit (Qiagen), labelled according to the manufacturer's instructions using the Quick-Amp One-Color Labelling Kit (Agilent Technologies) and hybridized to Agilent custom microarrays. Three replicates were performed for each genotype.

### Protein extraction and immuno blotting

Protein was extracted in buffer containing 50 mM Tris-HCl pH 7.5, 150 mM NaCl, 10% glycerol, Sigma Protease Inhibitor (CSD2) or 20 mM Tris-HCl pH 7.5, 300 mM NaCl, 5 mM MgCl_2_, 0.1% IGEPAL CA-630, 5 mM DTT, Sigma Protease Inhibitor (AGO1). Protein concentrations were determined using BioRad DC protein assay. Five µg (CSD2) and 80 ug (AGO1) of protein were resuspended in Laemmli buffer (20 mM Tris-HCl pH 6.8, 2% SDS, 5% glycerol, 40 mM DTT and 0.02% bromophenol blue), heated at 100°C for 5 minutes, and separated on a 15% (CSD2) or 6% (AGO1) SDS-PAGE gel. Proteins were transferred to PVDF membrane (BioRad). For detection, the membrane was blocked in 5% non-fat dry milk in 1×TBS, 0.1% Tween-20 (1×TBST) for 1 hour at room temperature, and incubated with a 1∶1000 dilution of CSD2 primary polyclonal antibody (Agrisera) or 1∶5000 dilution of AGO1 primary antibody ([33], Eurogenetech) in 5% non-fat dry milk and 1×TBST for 1.5 hours at room temperature. The membrane was then rinsed in 1×TBST for 45 minutes before incubation with a secondary peroxidase-conjugated anti-rabbit antibody (Sigma) in 5% non-fat dry milk in 1×TBST at room temperature for one hour. After the membrane was rinsed in 1×TBST for 45 minutes, CSD2 and AGO1 signals were revealed using the Western Lightning kit (PerkinElmer Life Sciences) kit and the Immunstar WesternC kit (Biorad) at the manufacturer's specifications.

### Microscopy and image analysis

For fluorescence studies, embryos where dissected from ovules using fine tip syringes in 10% glycerol, mounted on slides and analyzed using an AxioImager microscope (Zeiss) with YFP or CFP filter sets. Images were taken using Axiovision 4.4 software (Zeiss) and figures were generated using Photoshop 7.0 (Adobe). For confocal pictures, a Leica TCS SP2 AOBS spectral confocal microscope was used. Embryos were stained with DAPI (1 mg/ml) for 5 minutes and mounted in 50% glycerol in 1×PBS.

### Construction of fluorescent protein genes and chimeric genes

All *AGO1* and *ZLL* sequences for both the fluorescent protein fusion and chimeric constructs are derived from the Col accession. *AGO1* and *ZLL* chimeric constructs were made by exchanging five genomic domains; the 5′ sequence upstream of the ATG, the N-terminal, PAZ and the MID-PIWI domains and the 3′ region downstream of the stop codon. For cloning, restriction sites were introduced within introns at the appropriate positions (Table S4 and Figure S8). During the course of this work, we re-sequenced the *ZLL* L*er* gene and several new *ZLL* cDNA clones and found that the original report of six amino acid differences between the ZLL Col and ZLL L*er* proteins [15] was in error. The ZLL L*er* amino acid sequence is identical to that of ZLL in Col, as previously published [14].

## Supporting Information

Figure S1 Loss of *ZLL* function partially restores leaf development in *ago1-27*. (A) 20-day-old seedlings of Col and *zll^ago10-3^* and 24-day-old seedlings of *ago1-27*, *ago1-27* *zll^ago10-1^*, and *ago1-27* *zll^ago10-3^*. Serration of leaves is reduced markedly in both *ago1-27* *zll^ago10-1^* and *ago1-27* *zll^ago10-3^* compared with *ago1-27*. (B) RT-PCR was performed on RNA from Col and *zll^ago10-1^* inflorescences. Tubulin was used as control. Quantitative RT-PCR was performed on RNA from Col and *zll^ago10-3^* 12-day-old seedlings. Values were normalized to *EF1a* control.
(1.91 MB TIF)

Figure S2 ZLL and AGO1 act redundantly in embryogenesis. (A–D) Late globular stage. The formation of the lens-shaped cell (lsc marked in blue) is disturbed in segregating embryos of *ago1-1/+ zll^ago10-1^* plants where the hypophysis divides longitudinally instead of transversely, compared to wildtype and single mutants. Provascular cells stay isodiametric and do not elongate in putative double embryos (marked in red) compared to wildtype and single mutants. (E–H) Late torpedo stage. Putative double mutant embryos arrest development without initiating organs as spherical structures. Segregating *ago1-1* embryos display a broader apex and a wider angle between the cotyledons than wildtype or *zll* mutants. (I–L) Expression of WOX5:NLS-GFP in the QC cells and the upper suspensor cells is undetectable in putative double mutant embryos in contrast to wildtype and single mutants. (M–P) Expression of gWUS:GFP3 in the organizing center of the shoot meristem is expanded in putative double mutant embryos in comparison to wildtype and single mutants. Genotype of the mother plants and the percentage of embryos displaying the given phenotype are indicated. N: number of analyzed embryos; scale bar: 10 µm.
(2.07 MB TIF)

Figure S3 *zll* mutations enhance miR398-directed silencing of *CSD2* when AGO1 function is partially compromised. (A) RNA gel blot analysis of miR398 in the indicated mutant and control lines in the presence (0.5 µM) or absence (0 µM) of CuSO^4^. U6 hybridization was used as a loading control. Normalized values of miR398 to U6 RNA (with Col controls set at 1.0) are indicated. Immuno blot of CSD2 protein in the indicated mutant lines and their Col control in the presence (0.5 µM) or absence (0 µM) of CuSO^4^. Coomassie blue-stained RUBISCO small subunit (RbcS) serves as a loading control. (B) Quantitative RT-PCR of *CSD2* mRNA in the indicated mutant lines in the presence (0.5 µM, white bars) or absence (0 µM, black bars) of CuSO^4^. Average values of three technical replicates were normalized to *EF1a* control values and standard deviations are shown.
(0.22 MB TIF)

Figure S4 Loss of ZLL function in *ago1 hypomorphic mutants increases AGO1 protein levels. Immuno blot of AGO1 protein in inflorescences of the indicated mutant lines and their Col control. Biological replicates are shown for ago1-40zll-3^Col^ and ago1-40. Coomassie blue-stained RUBISCO small subunit (RbcS) serves as a loading control.*
(0.12 MB TIF)

Figure S5 Rescue of ago1 defects by AGO1^ZLL^ chimeras. 40-day-old wildtype Col, *ago1-27* and *ago1-1* mutant plants harboring chimeric constructs as indicated.
(4.55 MB TIF)

Figure S6 *ago1-1* seedlings expressing *pAGO1:ZLL*. (A) 20-day-old seedlings of *ago1-1* and *ago1-1/pAGO1:ZLL*. Radialization of the leaves is reduced in *ago1-1/pAGO1:ZLL* compared to ago1-1. (B) Quantification of leaf length per width in *ago1-1* and *ago1-1/pAGO1:ZLL* seedlings. n, number of analyzed plants. Plants were grown on 0,5×MS medium supplemented with 1% sucrose and Gamborg vitamins and then transferred to soil, which allows for limited leaf development in the *ago1-1* mutant.
(0.92 MB TIF)

Figure S7 Rescue of *ago1* defects by AGO1^ZLL^ chimeras. (A) The flowering times of Col, *ago1-27*, and *ago1-27* harboring the indicated constructs. Two to three independent lines per construct and eight plants per line were analyzed. (B) The rosette diameter of 24-day-old plants for Col, *ago1-27*, and *ago1-27* harboring the indicated constructs. Two to three independent T2 lines for each construct were analyzed. Standard deviations are given. The blue and orange dashed lines indicate the rosette diameter of Col and *ago1-27*, respectively. The dark grey and light grey boxes indicate rescued and non-rescued development, respectively.
(0.30 MB TIF)

Figure S8 Schematic of chimeric *AGO1* and *ZLL* constructs. (A) The fragments used and the introduced restriction sites are shown. Functional domains are indicated. (B) Genomic organization of the *ZLL* gene. (C) Genomic organization of the *AGO1* gene.
(0.21 MB TIF)

Table S1 Average GUS activity ± SE at different developmental stages.
(0.03 MB DOC)

Table S2 Transcript levels of miRNA targets significantly (p-value<0.05 or marked with * p value<0.01) upregulated in ago1-27 compared to wildtype, and downregulated in ago1-27 zll ago10-1 compared to ago1-27. n.s., no significant difference.
(0.05 MB DOC)

Table S3 Suppression of *ago1-27* defects by *pAGO1:CFP-AGO1*.
(0.02 MB DOC)

Table S4 Primer combinations and introduced restriction sites used to clone the chimeric *AGO1^ZLL^* genes.
(0.02 MB DOC)
