# Supplementary figures and images for: Redundant and Specific Roles of the ARGONAUTE Proteins AGO1 and ZLL in Development and Small RNA-Directed Gene Silencing
Source: PLoS Genet. 2009 Sep 18;5(9):e1000646. doi: 10.1371/journal.pgen.1000646 (PMC2730571; doi:10.1371/journal.pgen.1000646)

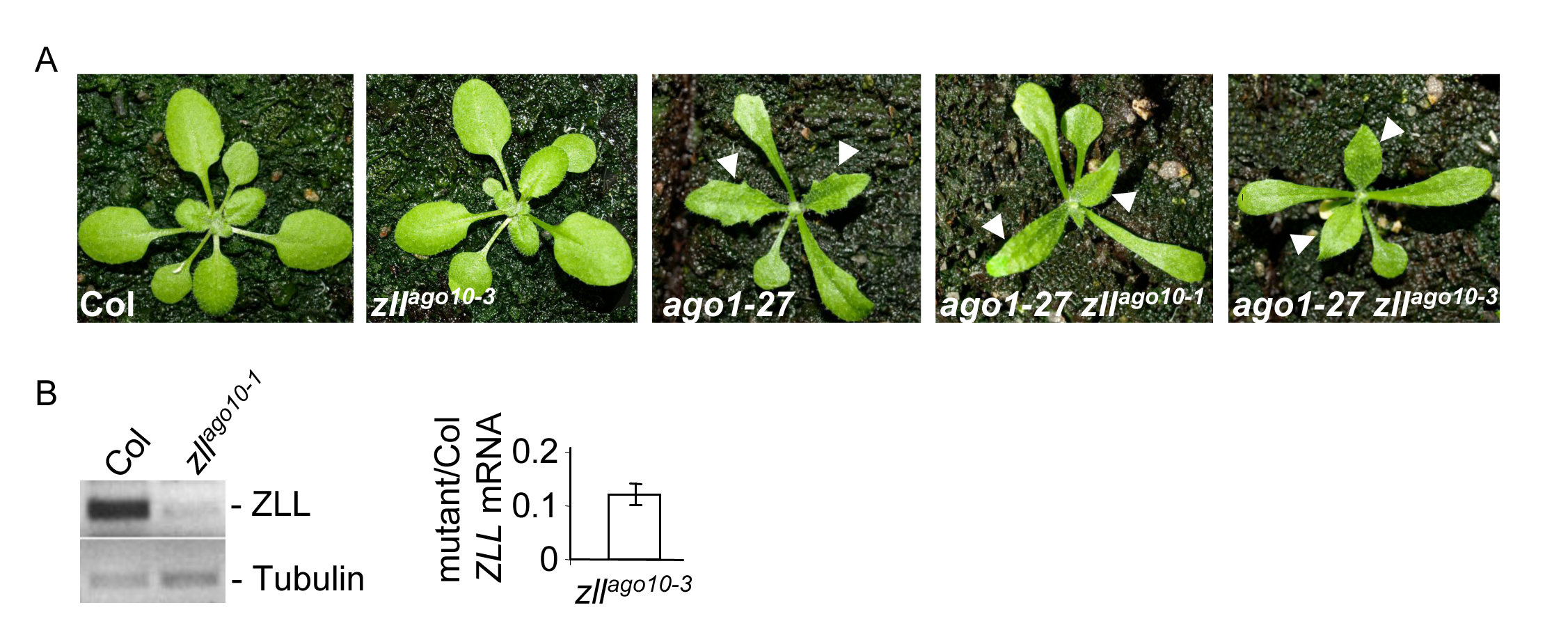

Supplement: Figure S1 — Loss of ZLL function partially restores leaf development in ago1-27. (A) 20-day-old seedlings of Col and zllago10-3 and 24-day-old seedlings of ago1-27, ago1-27 zllago10-1, and ago1-27 zllago10-3. Serration of leaves is reduced markedly in both ago1-27 zllago10-1 and ago1-27 zllago10-3 compared with ago1-27. (B) RT-PCR was performed on RNA from Col and zllago10-1 inflorescences. Tubulin was used as control. Quantitative RT-PCR was performed on RNA from Col and zllago10-3 12-day-old seedlings. Values were normalized to EF1a control. (1.91 MB TIF) [file pgen.1000646.s001.tif]

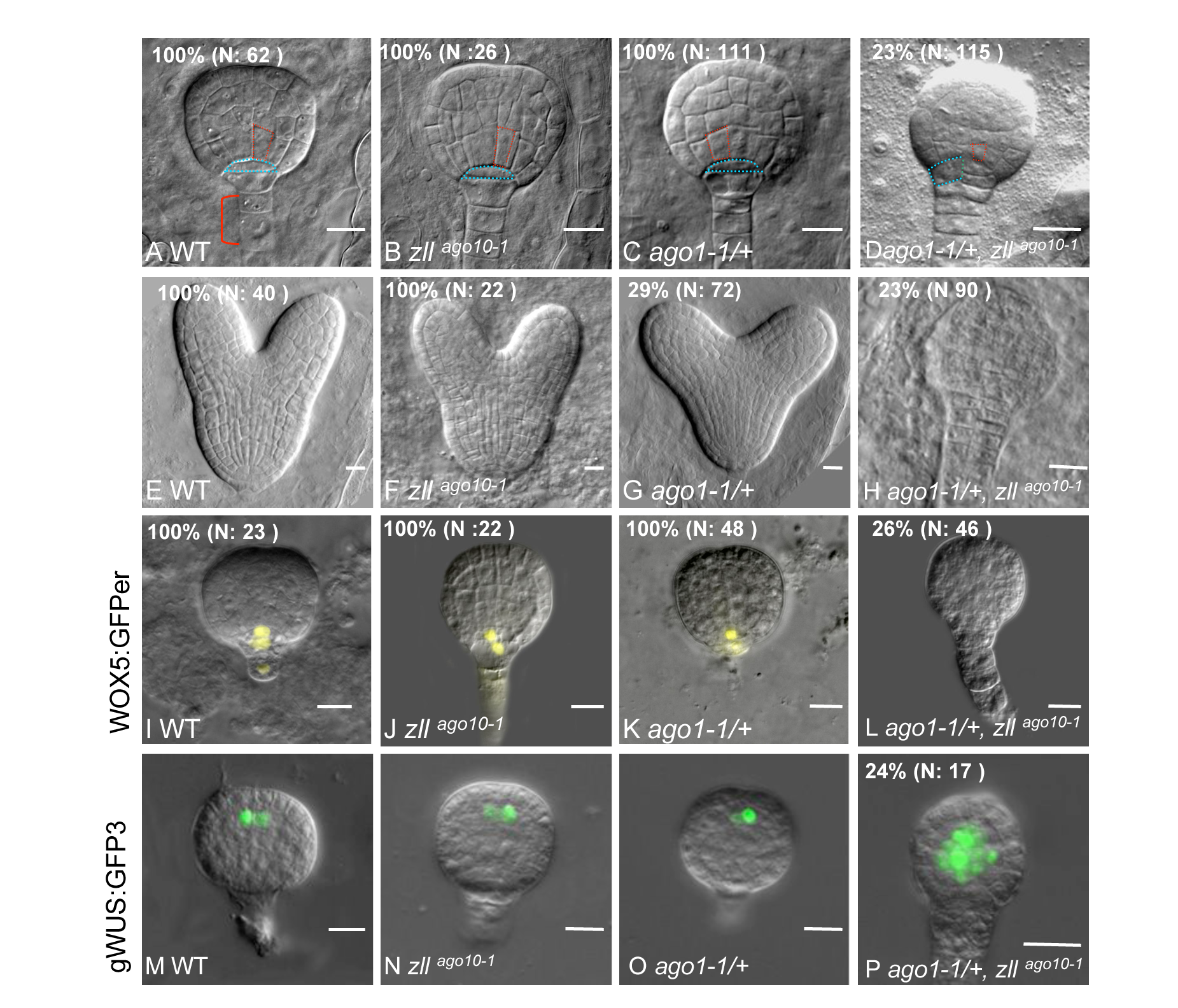

Supplement: Figure S2 — ZLL and AGO1 act redundantly in embryogenesis. (A–D) Late globular stage. The formation of the lens-shaped cell (lsc marked in blue) is disturbed in segregating embryos of ago1-1/+ zllago10-1 plants where the hypophysis divides longitudinally instead of transversely, compared to wildtype and single mutants. Provascular cells stay isodiametric and do not elongate in putative double embryos (marked in red) compared to wildtype and single mutants. (E–H) Late torpedo stage. Putative double mutant embryos arrest development without initiating organs as spherical structures. Segregating ago1-1 embryos display a broader apex and a wider angle between the cotyledons than wildtype or zll mutants. (I–L) Expression of WOX5:NLS-GFP in the QC cells and the upper suspensor cells is undetectable in putative double mutant embryos in contrast to wildtype and single mutants. (M–P) Expression of gWUS:GFP3 in the organizing center of the shoot meristem is expanded in putative double mutant embryos in comparison to wildtype and single mutants. Genotype of the mother plants and the percentage of embryos displaying the given phenotype are indicated. N: number of analyzed embryos; scale bar: 10 µm. (2.07 MB TIF) [file pgen.1000646.s002.tif]

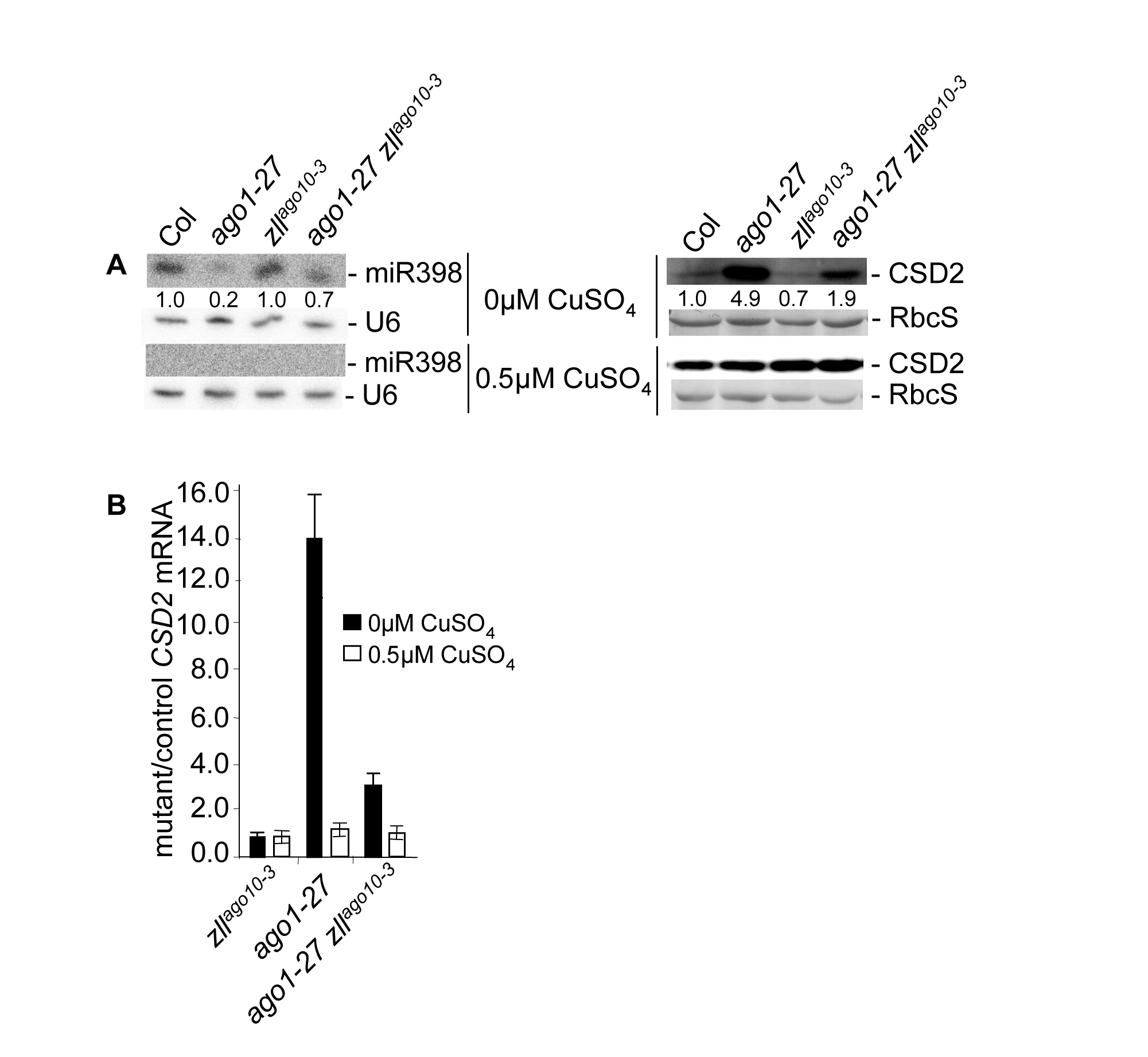

Supplement: Figure S3 — zll mutations enhance miR398-directed silencing of CSD2 when AGO1 function is partially compromised. (A) RNA gel blot analysis of miR398 in the indicated mutant and control lines in the presence (0.5 µM) or absence (0 µM) of CuSO4. U6 hybridization was used as a loading control. Normalized values of miR398 to U6 RNA (with Col controls set at 1.0) are indicated. Immuno blot of CSD2 protein in the indicated mutant lines and their Col control in the presence (0.5 µM) or absence (0 µM) of CuSO4. Coomassie blue-stained RUBISCO small subunit (RbcS) serves as a loading control. (B) Quantitative RT-PCR of CSD2 mRNA in the indicated mutant lines in the presence (0.5 µM, white bars) or absence (0 µM, black bars) of CuSO4. Average values of three technical replicates were normalized to EF1a control values and standard deviations are shown. (0.22 MB TIF) [file pgen.1000646.s003.tif]

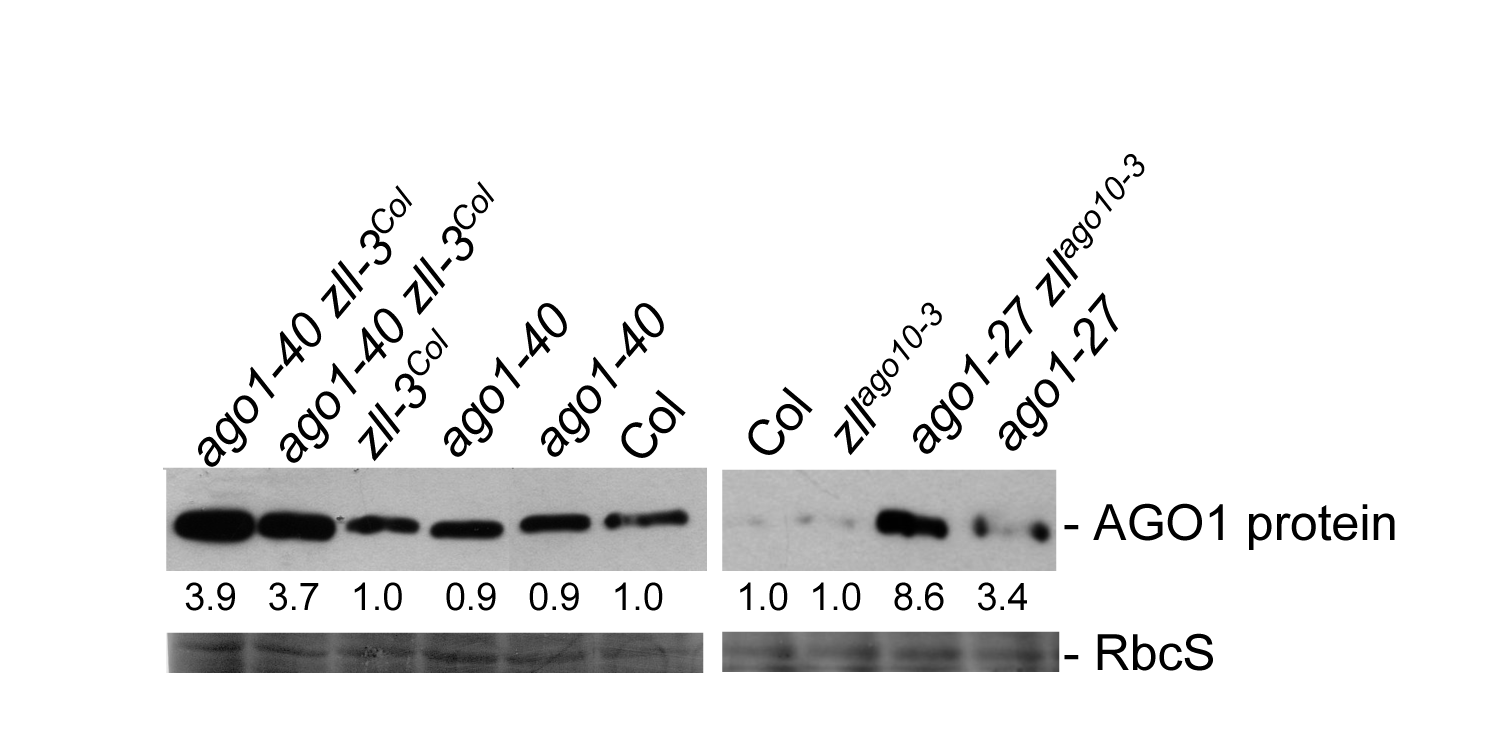

Supplement: Figure S4 — Loss of ZLL function in ago1 hypomorphic mutants increases AGO1 protein levels. Immuno blot of AGO1 protein in inflorescences of the indicated mutant lines and their Col control. Biological replicates are shown for ago1-40zll-3Col and ago1-40. Coomassie blue-stained RUBISCO small subunit (RbcS) serves as a loading control. (0.12 MB TIF) [file pgen.1000646.s004.tif]

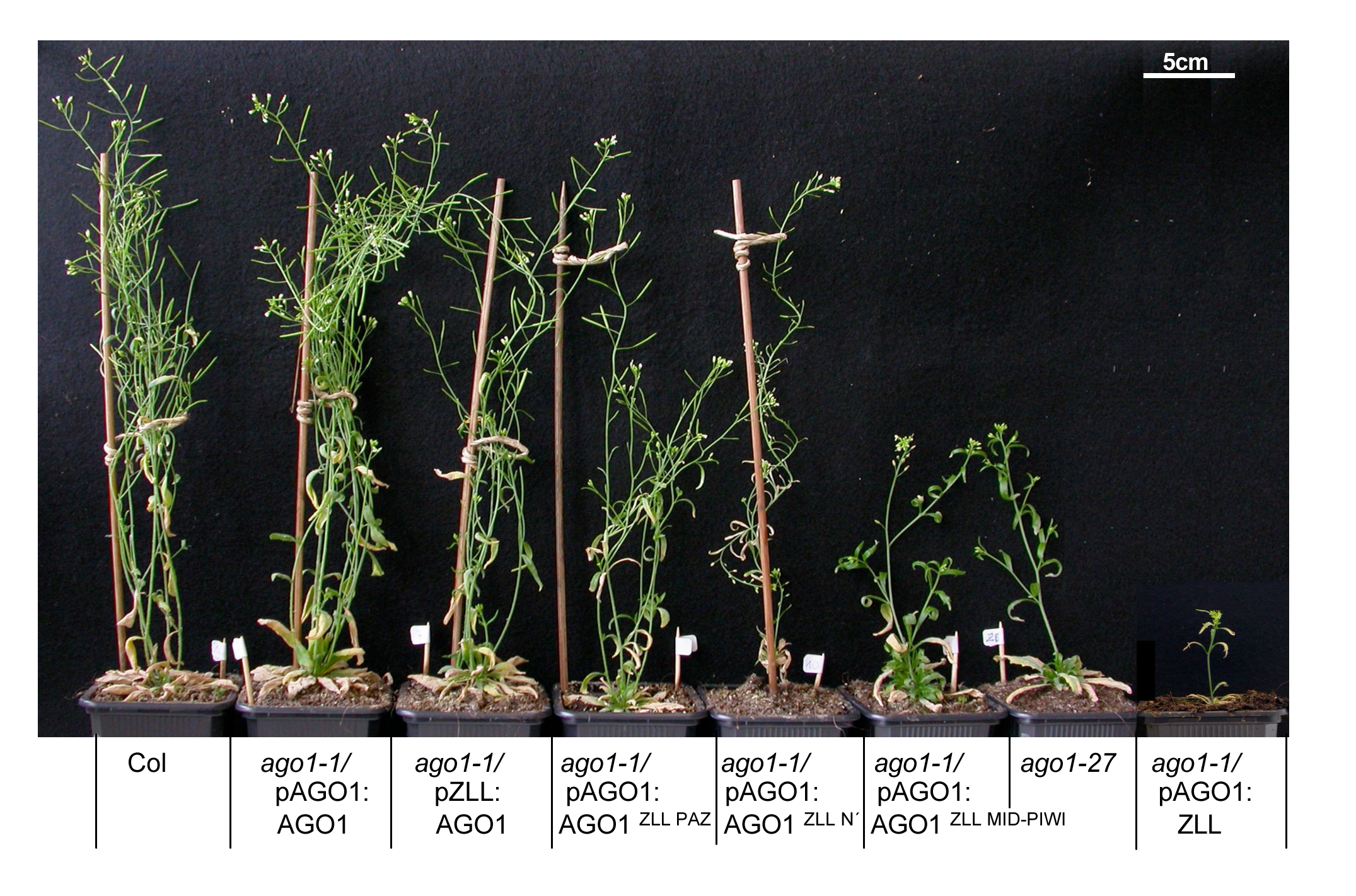

Supplement: Figure S5 — Rescue of ago1 defects by AGO1ZLL chimeras. 40-day-old wildtype Col, ago1-27 and ago1-1 mutant plants harboring chimeric constructs as indicated. (4.55 MB TIF) [file pgen.1000646.s005.tif]

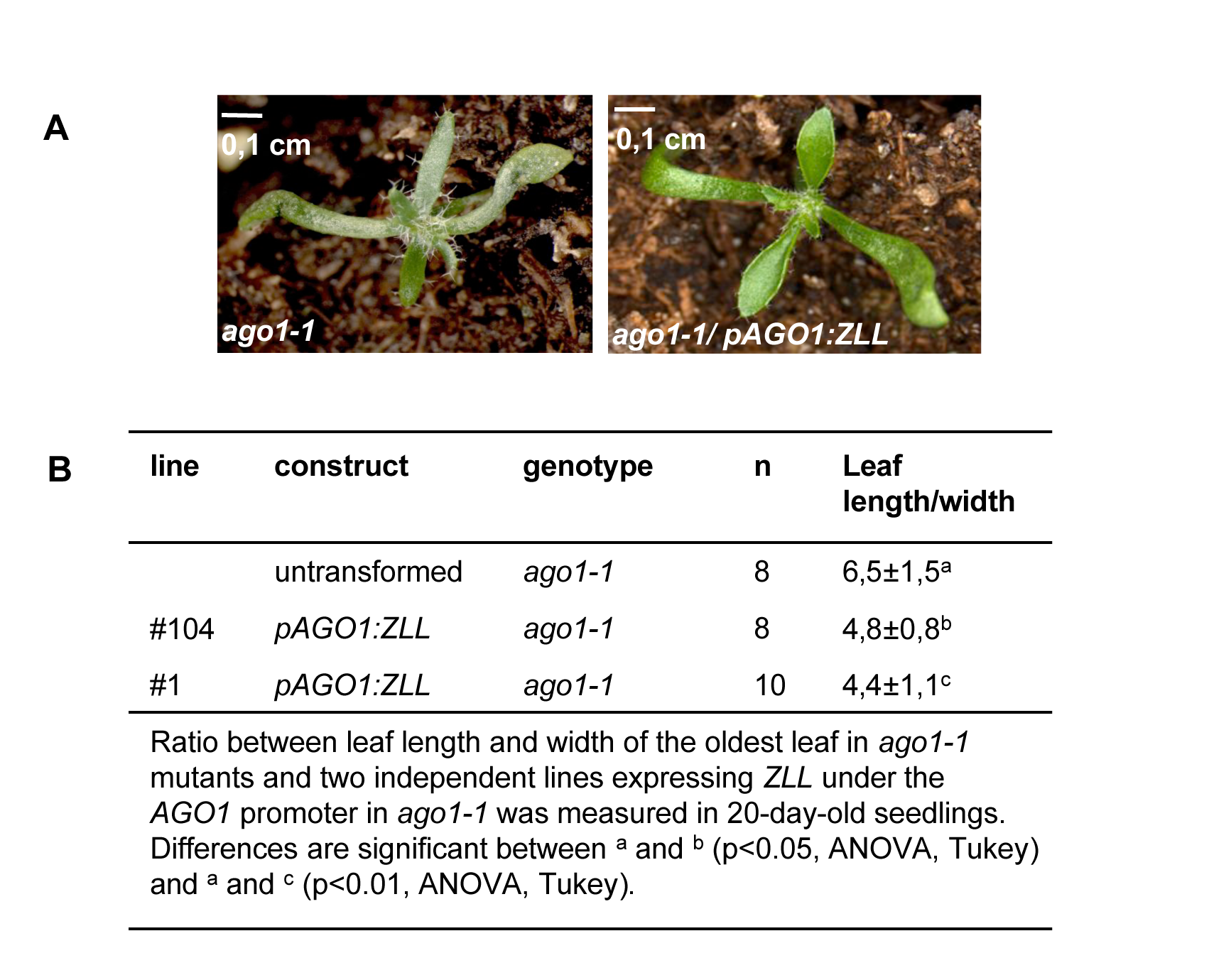

Supplement: Figure S6 — ago1-1 seedlings expressing pAGO1:ZLL. (A) 20-day-old seedlings of ago1-1 and ago1-1/pAGO1:ZLL. Radialization of the leaves is reduced in ago1-1/pAGO1:ZLL compared to ago1-1. (B) Quantification of leaf length per width in ago1-1 and ago1-1/pAGO1:ZLL seedlings. n, number of analyzed plants. Plants were grown on 0,5×MS medium supplemented with 1% sucrose and Gamborg vitamins and then transferred to soil, which allows for limited leaf development in the ago1-1 mutant. (0.92 MB TIF) [file pgen.1000646.s006.tif]

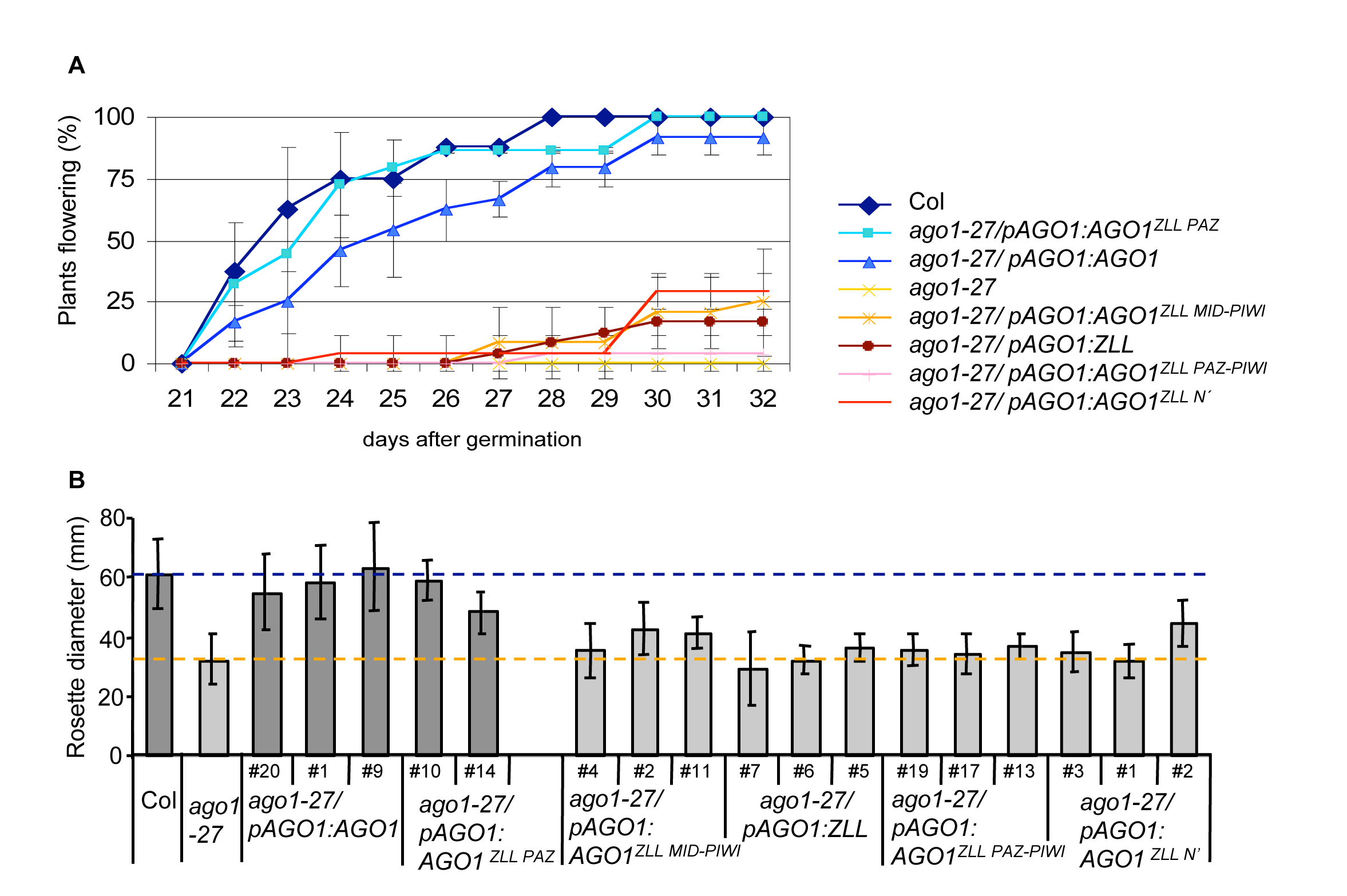

Supplement: Figure S7 — Rescue of ago1 defects by AGO1ZLL chimeras. (A) The flowering times of Col, ago1-27, and ago1-27 harboring the indicated constructs. Two to three independent lines per construct and eight plants per line were analyzed. (B) The rosette diameter of 24-day-old plants for Col, ago1-27, and ago1-27 harboring the indicated constructs. Two to three independent T2 lines for each construct were analyzed. Standard deviations are given. The blue and orange dashed lines indicate the rosette diameter of Col and ago1-27, respectively. The dark grey and light grey boxes indicate rescued and non-rescued development, respectively. (0.30 MB TIF) [file pgen.1000646.s007.tif]

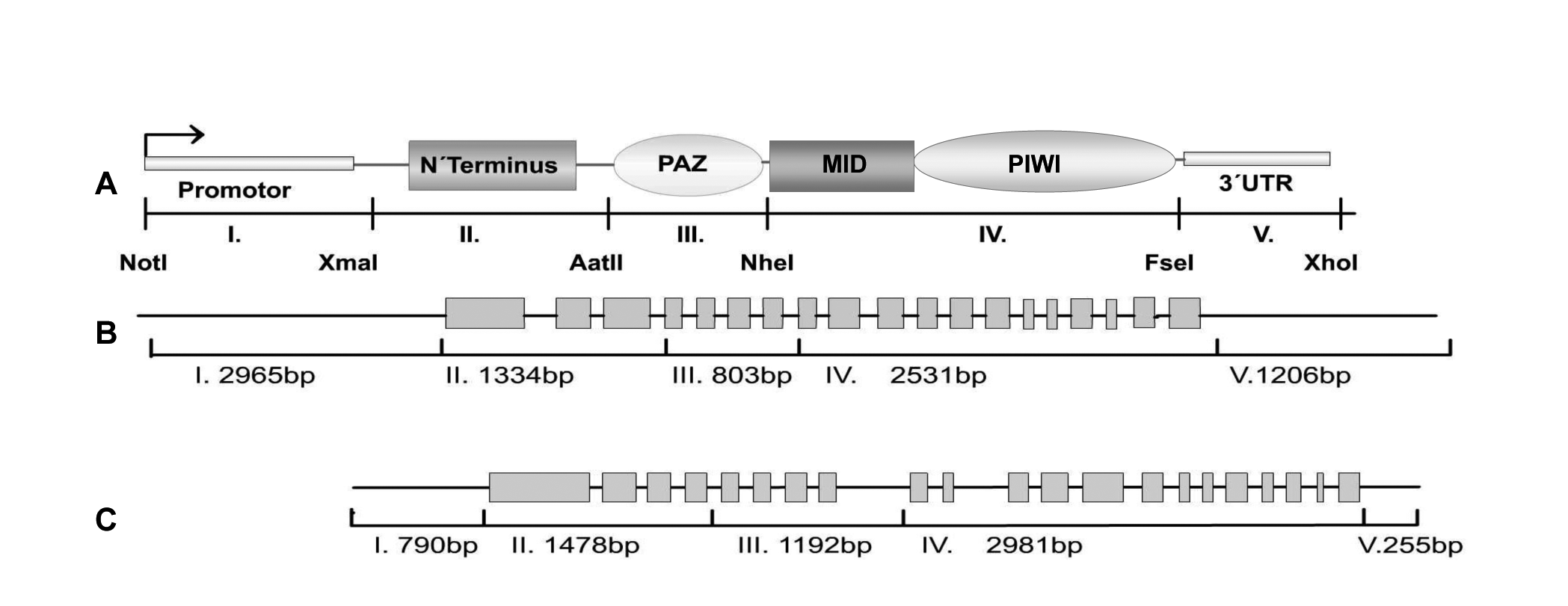

Supplement: Figure S8 — Schematic of chimeric AGO1 and ZLL constructs. (A) The fragments used and the introduced restriction sites are shown. Functional domains are indicated. (B) Genomic organization of the ZLL gene. (C) Genomic organization of the AGO1 gene. (0.21 MB TIF) [file pgen.1000646.s008.tif]
