# Supplementary material for: Redundant and Specific Roles of the ARGONAUTE Proteins AGO1 and ZLL in Development and Small RNA-Directed Gene Silencing
Source: PLoS Genet. 2009 Sep 18;5(9):e1000646. doi: 10.1371/journal.pgen.1000646 (PMC2730571; doi:10.1371/journal.pgen.1000646)
Supplement: Table S1 — Average GUS activity ± SE at different developmental stages. (0.03 MB DOC) [file pgen.1000646.s009.doc]

**Supplementary Table 1:** Average GUS activity **±** SE at different developmental stages.

| Plant Genotype | 7-day-old seedlings^a^ | 15-day-old seedlings^a^ | Rosette leaf | Inflorescence | Percentage of silenced plants^b^ |
| --- | --- | --- | --- | --- | --- |
| L1 | 9.0 ± 1.0 | 18.0 ± 4.9 | 3.0 ± 0.8 | 0 | 100% |
| *L1/zll-3*^Col^ | 6.0 ± 0.6 | 5.0 ± 0.4 | 1.0 ± 0.4 | 0 | 100% |
| *L1/ago1-40* | 1321 ± 173.8 | 598.0 ± 88.2 | 1938 ± 194.7 | 624.0 ± 79.8 | 50% |
| *L1/ago1-40/zll-3*^Col^ | 198.0 ± 32.8 | 154.0 ± 43.4 | 159.0 ± 38.7 | 6.8 ± 2.0 | 94% |
| ^a^ Does not include roots. ^b^ Inflorescence. n = 48 for all genotypes. Average GUS protein activity (nmol MU/min/mg protein) is reported. SE, standard error. At all four developmental stages, the *L1/ago1-40* GUS values are significantly different from the *L1* control GUS values and the *L1/ago1-40/zll-3^Col^* GUS values are significantly different from the *L1/ago1-40* GUS values (Student’s t-test). | | | | | |
