# Supplementary material for: Redundant and Specific Roles of the ARGONAUTE Proteins AGO1 and ZLL in Development and Small RNA-Directed Gene Silencing
Source: PLoS Genet. 2009 Sep 18;5(9):e1000646. doi: 10.1371/journal.pgen.1000646 (PMC2730571; doi:10.1371/journal.pgen.1000646)
Supplement: Table S2 — Transcript levels of miRNA targets significantly (p-value<0.05 or marked with * p value<0.01) upregulated in ago1-27 compared to wildtype, and downregulated in ago1-27 zll ago10-1 compared to ago1-27. n.s., no significant difference. (0.05 MB DOC) [file pgen.1000646.s010.doc]

**Supplementary Table 2:** Transcript levels of miRNA targets significantly

(p-value <0.05 or marked with * p value<0.01) upregulated in *ago1-27* compared

to wildtype, and downregulated in *ago1-27* *zll ^ago10-1^* compared to  *ago1-27*. n.s., no significant difference.

|  |  | Fold changes | | |  |
| --- | --- | --- | --- | --- | --- |
| **Name** | **Locus name** | *ago1-27*  vs wt | *zll ^ago10-1^*  vs wt | *ago1-27 zll^ago10-1^* vs  *ago1-27* | **Targeting miRNA [1-5]** |
| CUC2 | AT5G53950 | 4.17 * | n.s. | -1.49* | miR164 |
| unknown | AT5G28520 | 1.86* | n.s. | -1.45 | miR846 |
| ATUBC24 | AT2G33770 | 1.51* | n.s. | -1.29 | miR399 |
| DCL1 | AT1G01040 | 1.75* | n.s. | -1.57* | miR162 |
| PPR | AT1G63230 | 1.47* | n.s. | -1.33 | miR161 |
| ATCHX18 | AT5G41610 | 1.89* | n.s. | -1.71 | miR780, miR856 |
| Disease R. | AT5G43730 | 1.48 | n.s. | -1.38 | miR472 |
| EMB2745 | AT5G39710 | 1.34 | n.s. | -1.25 | miR400 |
| AtGRF8 | AT4G24150 | 1.45 | n.s. | -1.40 | miR396 |
| ARF17 | AT1G77850 | 1.57* | n.s. | -1.53* | miR160 |
| PHV | AT1G30490 | 1.49* | n.s. | -1.53* | miR165/166 |
| PPR | AT5G16640 | 1.35 | n.s. | -1.40 | miR161, miR400 |
| REV | AT5G60690 | 1.36 | n.s. | -1.42* | miR165/166 |
| CMT3 | AT1G69770 | 1.45 | n.s. | -1.53 | miR823 |
| ARF10 | AT2G28350 | 1.58* | n.s. | -1.76* | miR160 |
| TAS3 | AT3G17185 | 1.60 | n.s. | -1.88* | miR390 |
| F-box | AT2G24510 | 1.48 | n.s. | -1.79* | miR859 |
|  | AT5G23480 | 1.66* | n.s. | -2.03* | miR781 |
| PHB | AT2G34710 | 1.40 | n.s. | -1.80* | miR165/166 |
| ATHB-15 | AT1G52150 | 1.37 | n.s. | -1.93* | miR165,/166 |

**Supplemental References**

1. Fahlgren N, Howell MD, Kasschau KD, Chapman EJ, Sullivan CM, et al. (2007) High-throughput sequencing of Arabidopsis microRNAs: evidence for frequent birth and death of MIRNA genes. PLoS One 2: e219.

2. Jones-Rhoades MW, Bartel DP (2004) Computational identification of plant microRNAs and their targets, including a stress-induced miRNA. Mol Cell 14: 787-799.

3. Lu C, Kulkarni K, Souret FF, MuthuValliappan R, Tej SS, et al. (2006) MicroRNAs and other small RNAs enriched in the Arabidopsis RNA-dependent RNA polymerase-2 mutant. Genome Res 16: 1276-1288.

4. Rajagopalan R, Vaucheret H, Trejo J, Bartel DP (2006) A diverse and evolutionarily fluid set of microRNAs in Arabidopsis thaliana. Genes Dev 20: 3407-3425.

5. Rhoades MW, Reinhart BJ, Lim LP, Burge CB, Bartel B, et al. (2002) Prediction of plant microRNA targets. Cell 110: 513-520.
