# Supplementary material for: Redundant and Specific Roles of the ARGONAUTE Proteins AGO1 and ZLL in Development and Small RNA-Directed Gene Silencing
Source: PLoS Genet. 2009 Sep 18;5(9):e1000646. doi: 10.1371/journal.pgen.1000646 (PMC2730571; doi:10.1371/journal.pgen.1000646)
Supplement: Table S3 — Suppression of ago1-27 defects by pAGO1:CFP-AGO1. (0.02 MB DOC) [file pgen.1000646.s011.doc]

**Supplementary Table 3:** Suppression of *ago1-27* defects by *pAGO1:CFP-AGO1.*

| **Developmental stage** | **Phenotypes of transformed T1 plants (%)** | |
| --- | --- | --- |
|  | wildtype-like | *ago1-27*-like |
| seedling | 85 | 15 |
| adult | 50 | 50 |
| Plants where categorized as "*ago1-27* like" if they displayed narrow and small leaves, a delay in flowering time or reduction in rosette size compared to wildtype.  100 seedlings were analyzed for each experiment. | | |
