# Supplementary material for: Redundant and Specific Roles of the ARGONAUTE Proteins AGO1 and ZLL in Development and Small RNA-Directed Gene Silencing
Source: PLoS Genet. 2009 Sep 18;5(9):e1000646. doi: 10.1371/journal.pgen.1000646 (PMC2730571; doi:10.1371/journal.pgen.1000646)
Supplement: Table S4 — Primer combinations and introduced restriction sites used to clone the chimeric AGO1ZLL genes. (0.02 MB DOC) [file pgen.1000646.s012.doc]

**Supplementary Table 4:** Primer combinations and introduced restriction sites used to clone the chimeric *AGO1^ZLL^* genes.

| **Gene** | **Fragment** | **Primer** | **Adapter** |
| --- | --- | --- | --- |
| ZLL | Promoter | gcggccgcggcgcgccAGGCCGGTGGTTTGCA  CCCCCCGGGGGGTTTGGATTTTCAAAAACTCT | NotI/AscI  XmaI |
| ZLL | N´ terminus | CCCCCCGGGGGGCAACAAAAAATGCCGATTAG  CCGCTCGAGTAGACGTCTTGGGCTGACTTATAAAATA | XmaI  AatII/XhoI |
| ZLL | PAZ | GACGTCTCTTAATGTTATTCTCCTTACTTT  CCGCTCGAGTAGCTAGCGTTCTAACTCACAAAGAACC | AatII  NheI/XhoI |
| ZLL | MID-PIWI | CTAGCTAGCTTAGAAGTGAGGATTAATCTTG  CCGCTCGAGTAGGCCGGCCTGGATTTTTAGCAGTAGAAC | NheI  FseI/XhoI |
| ZLL | 3´UTR | GGCCGGCCAACATTCCTTAATCAGTTTT  CTCGAGGTGCGGTATACTGCAGGTTAATA | FseI  XhoI |
| AGO1 | Promoter | ATAAGAATGCGGCCGCAGGCGCGCCTCGTCGACATATGCCAGCTAT  CCCCCCGGGGGGCCTGTGAAAATAACACAACC | NotI/AscI  XmaI |
| AGO1 | N´ terminus | CCCCCCGGGGGGACAGGAATCATCATGGTGAG  CCGCTCGAGTAGACGTCCCATGAGTTAAACATTCAAG | XmaI  AatII/XhoI |
| AGO1 | PAZ | GACGTCTTGTGCTGCAATTTTGTCTG  CCGCTCGAGTAGCTAGCATAAACAATTGAGTTTTCAA | AatII  NheI/XhoI |
| AGO1 | MID-PIWI | CTAGCTAGCTGCTAACATTATACAATATTTTC  CCGCTCGAGTAGGCCGGCCTCAACTCAGCAGTAGAACAT | NheI  FseI/XhoI |
| *AGO1* | 3´UTR | CAGGCCGGCCTTCACCCTCTATCTATCTTTATGAC  CCGCTCGAGTATGTGGTCGACAATACAAACA | FseI  XhoI |
